# Fibroblast growth factor receptor 1-bound extracellular vesicle as novel therapy for osteoarthritis

**DOI:** 10.37796/2211-8039.1308

**Published:** 2022-06-01

**Authors:** Bryan Gervais de Liyis, John Nolan, Made Agus Maharjana

**Affiliations:** a Medical Faculty, Udayana University, Bali, Indonesia; b Department of Orthopaedic and Traumatology, Sanglah General Hospital Denpasar, Bali, Indonesia

**Keywords:** Extracellular vesicle, Fibroblast growth factor receptor 1, Osteoarthritis

## Abstract

Osteoarthritis (OA) is a joint condition that causes significant impairment of the chondrocyte. The gradual degradation of the cartilage lining of one or more freely moving joints, as well as persistent inflammation, are the causes of osteoarthritis. Current medications focus on alleviating symptoms rather than curing the condition. The aim of this review is to evaluate the potential use of fibroblast growth factor receptor 1-bound extracellular vesicle as novel therapy for osteoarthritis. This review article was completed by searching for the keywords “Fibroblast Growth Factor Receptor 1”, “Extracellular Vesicle”, and “Osteoarthritis” in various journals in several search engines. Of the 102 scientific articles found, 95 were found suitable to be used as material in the making of this article. The upregulated amount of FGFR1 (fibroblast growth factor receptors) signalling suggesting the progression of degenerative cartilage that commonly seen in osteoarthritis (OA) patients. Several studies showed that the involvement of extracellular vesicles (EV) derived from MSCs could enhance cartilage repair and protect the cartilage from degradation. EVs have the potential to deliver effects to specific cell types through ligand-receptor interactions and several pathway mechanisms related with the FGFR1. EVs and FGFR1-bound Evs have been postulated in recent years as possible therapeutic targets in human articular cartilage. The protective benefits on both chondrocytes and synoviocytes in OA patients can be achieved by administering the MSC-EVs that may also stimulate chondrocyte proliferation and migration EVs have a promising potential to become a novel therapy for treating patients with OA. However, further researches are need to be conducted to discover further the application of this therapy.

## 1. Introduction

Osteoarthritis (OA) is the most prevalent joint condition that causes significant impairment in a huge percentage of elderly people [1]. There are about 100 distinct kinds of arthritis, with OA being the most prevalent [2]. OA is a multifaceted and complex illness that may be described as persistent joint dysfunction affecting the whole joint [3]. In 2020, there were approximately 86.7 million people aged 20 and over with reported knee OA worldwide [4]. Calculation by the IHME GBD Tool suggests that the peak incidence of OA among 60 to 64 year-old is 1216 per 100,000 [5]. Furthermore, approximately 15.1 million people have symptomatic knee OA, with a lifetime risk of 13.8% [6]. The knee is the most prevalent location of OA in clinical practice, followed by the hand and hip [7]. The cause of osteoarthritis is the progressive degeneration of the cartilage lining of one or more freely moving joints and chronic inflammation [8,9]. This frequently results in incapacitating dysfunction, which can include different degrees of persistent pain, joint stiffness and edema, physical deconditioning, and a variety of functional, social, and vocational problems and limits [10]. Moreover, the risk factors of OA include obesity, traumas, advancing age, female sex and heredity [11]. There are now also substantial evidences that OA is a risk factor for the development of cardiovascular disease [12], memory loss [13] and diabetes [14].

Changes in extracellular matrix (ECM) composition or changes in the biomechanical environment of chondrocytes greatly enhance the risk of OA by disrupting signals important in the maintenance of normal cartilage development and homeostasis [15]. The discovery of prospective treatment targets implicated in OA pain or structural progression has been made possible by advances due to the knowledge of OA pathophysiology [2]. The pathophysiology of OA includes cartilage degradation and bone remodeling as a result of an active reaction of chondrocytes in the articular cartilage and inflammatory cells in the surrounding tissues [16]. The primary change is thought to be the loss of articular cartilage, but secondary changes include subchondral bone remodeling, the formation of osteophytes, the progression of bone marrow lesions, alteration in the synovium, joint capsule, ligaments and meniscal tears due to a combination of cellular changes and biomechanical stresses [17].

**Fig. 1:**
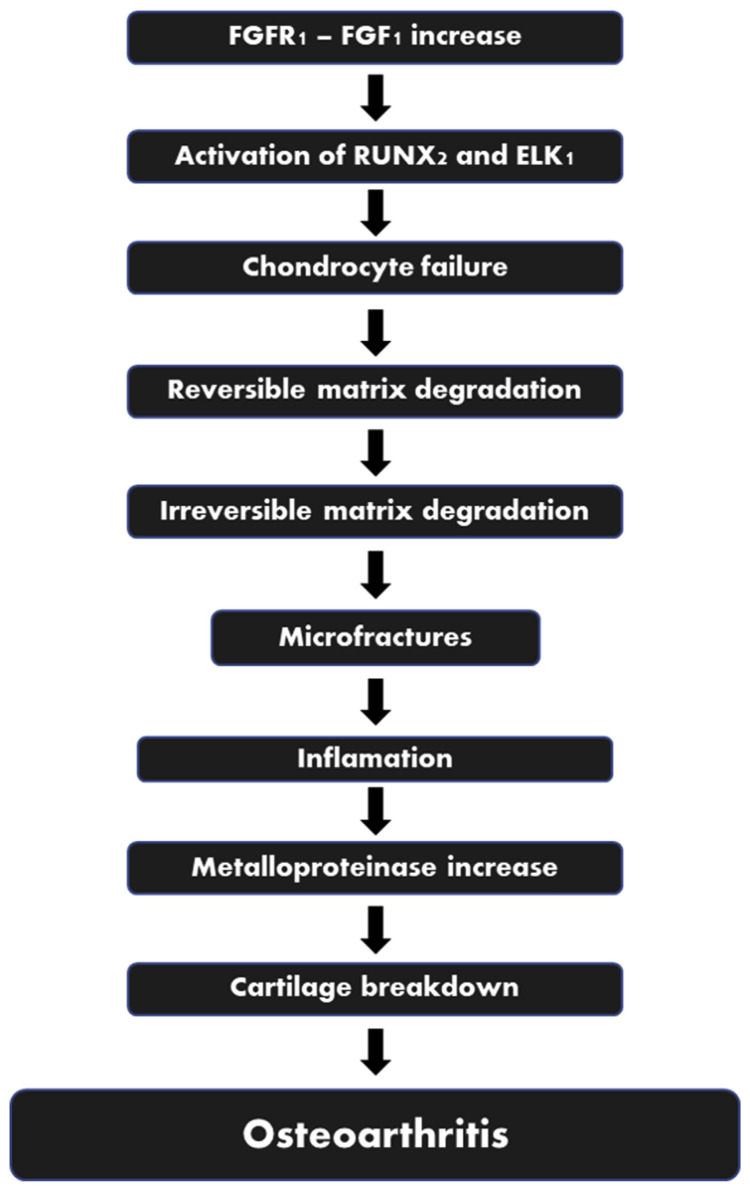
The Role of FGF1/FGFR1 in the Process of OA. Legend: RunX2: Runt-related transcription factor 2; ELK: ETS Like-1 protein.

Adult articular cartilage is composed of extracellular matrix (water, collagen and proteoglycans) and chondrocytes [18]. The regular turnover of the extracellular matrix components is governed by the chondrocytes that synthesize proteins and the proteolytic enzymes that break themdown [19]. Chondrocytes, in turn, are affected by a variety of variables, including polypeptide growth factors and cytokines, structural and physical stimulation, and even matrix components [20]. Multiple inflammatory mediators have been found in the synovial fluid of patients with OA, including plasma proteins (C-reactive protein), prostaglandins (PGE2), leukotrienes (LKB4), cytokines (TNF, IL1, IL6, IL15, IL17, IL18, IL21) and growth factors (TGF, FGFs, VEGF, NGF) [21]. One of the growth factor receptors, FGFR1, has catabolic effects inhuman articular chondrocytes and invertebrate disc tissue by upregulating matrix-degrading enzyme production, inhibiting ECM accumulation and proteoglycan synthesis, and clustering of cells, all of which are associated with arthritic conditions (Fig. 1) [22]. A significantly increased levels of FGFR1 is detected in both the chondrocytes, subchondral bone and synovium of OA patients [23,24]. Through the stimulation of RUNX2 and ELK1, FGFR1 promotes catabolic effects by limiting ECM synthesis and upregulating matrix-degrading enzyme production [25].

While there is no cure for OA, there are treatments that can help control symptoms and improve quality of life [26]. Currently, non-steroidal anti-inflammatory (NSAIDs) medications, analgesics including opioids, and intraarticular corticosteroids are among the conventional pharmacological treatments [27]. These therapy methods help alleviate arthritis symptoms but do not cure or inhibit the causal pathway of degeneration [28]. Although NSAIDs have a clinically significant therapeutic impact on OA pain, the benefits must be balanced against the risks such as cardiovascular, immunity and gastrointestinal complications [29,30]. Novel regenerative treatments have received a great deal of interest in recent years. In recent years, fibroblast growth factor (FGF) signalling has been implicated in cartilage homeostasis [31].

Fibroblast Growth Factor Receptors (FGFRs) are a group of receptor tyrosine kinases that are expressed on cell membranes and play important functions in the development of cells when bind with the corresponding Fibroblast Growth Factor (FGF) [32]. The human FGF gene family may be classified into eight subfamilies based on phylogenetic analysis: FGF1, FGF3, FGF4, FGF7, FGF8, FGF9, FGF11, and FGF19 [33]. FGFR1, FGFR2, and FGFR3 are the most numerous in the joint, with FGFR1 and FGFR3 being the most common receptors in human chondrocytes [34]. In degenerative cartilage of OA, the level of FGFR1 is increased relative to FGFR3, suggesting that FGFR1 is the main FGF route in cartilage degeneration [35]. Furthermore, conditional deletion of FGFR1 in a temporomandibular joint OA model has been shown to slow the development of the disease, and that inhibition of FGFR1 signalling may increase autophagic activity [23]. A novel therapy method purposed is to administer FGFR1-bound Extracellular Vesicles (EVs) to bind with the body’s FGF1, thus preventing binding with the body’s FGFR1.

Acknowledging the potential of FGFR1-bound EVs, the authors are interested in studying further regarding this modality so that it can provide better prospects in the management of osteoarthritis.

## 2. Method and materials

A literature review was utilized as the review approach. The literature references are from reputable search engines PubMed and Science Direct, and include terms like “Fibroblast Growth Receptor”, “Extracellular Vesicles,” and “Osteoarthritis”. All research linked to Fibroblast Growth Receptor 1 and Osteoarthritis are suitable for use as reference. At least five years should have passed since the studies were conducted. From the 102 journals examined, 95 were judged to be suitable for use as references in this work. The evaluated information is compiled into a single scientific literature review once it has been reviewed for credibility and dependability.

## 3. Results and discussion

### 3.1. Pathophysiology of osteoarthritis

**Fig. 2:**
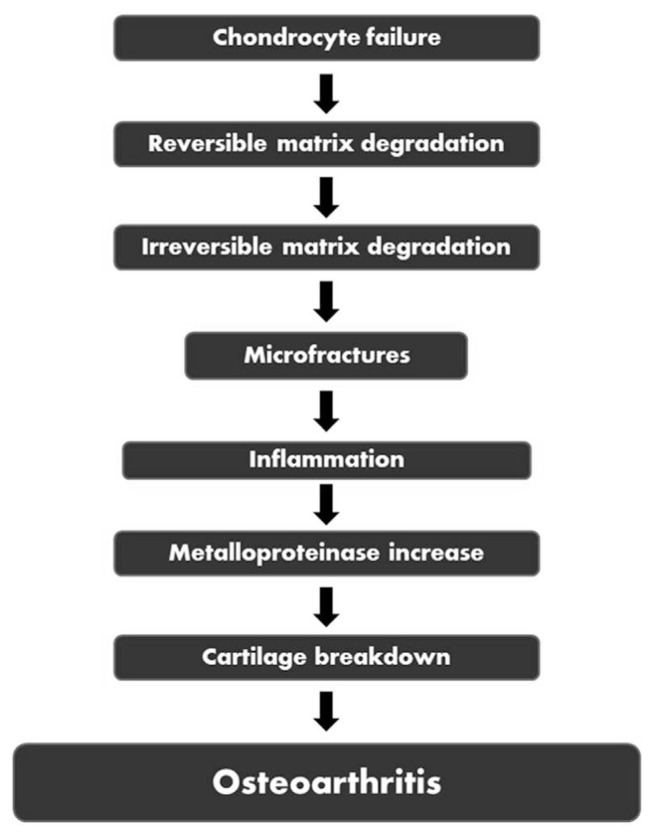
Pathophysiology of osteoarthritis.

OA is caused by the inability of chondrocytes to maintain equilibrium between the production and breakdown of these extracellular matrix components (Fig. 2) [36]. Trauma induces microfractures or inflammations that cause an increase in enzymatic activity leading to the production of “wear” particles and subsequently be ingested by local macrophages [20,37]. When the formation of these “wear” particles outweighs the system’s capacity to eliminate them, they become mediators of inflammation, causing the chondrocyte to produce degradative enzymes [19,20,36]. Proinflammatory cytokines such as TNF, IL-1, and IL-6 are also released when molecules from collagen and proteoglycan degradation are taken up by synovial macrophages [21,38]. These cytokines can attach to chondrocyte receptors, causing more metalloproteinases to be released and type II collagen synthesis to be inhibited, eventually accelerating cartilage breakdown [38]. Significant accumulation of adipokines in obese patients may also trigger the release of several inflammatory cytokines and proteases such as MMP-1, MMP-3, MMP-13, ADAMTS-4, TNF, IL1, IL6. These inflammatory mediators may further suppress the proliferation of chondrocytes in the cartilage and interference with the equilibrium between osteoblast and osteoclast in the bone [39].

### 3.2. FGFR1 expression in osteoarthritis

Related with the pathophysiology of OA, FGFRs were thought to be involved as FGF ligands played a major role in the conservation of articular cartilage. FGFRs in human joints are reported to play a significant role in the homeostasis of articular cartilage. In specific, FGFR1 is discovered to be eminently expressed in the articular cartilage of the knee [40]. Recent studies reported an escalating number of FGFR1 expressions along with a diminishing amount of FGFR3 found in the articular cartilage of OA patients. These expressions were exemplified in the mice models spontaneously and following the surgical procedure [41]. This suggests that the signalling of FGFR1 could accelerate the degradation of the matrix in articular cartilage.

The signalling of FGFR1 may promote the transcription factors expression of RUNX2 and ELK1. Expression of RUNX2 and ELK1 implicates the p38 MAPK and RAF–MEK–ERK pathways involvement [22]. Delayed FGFR1 signalling inhibits the catabolic response indicated by the decelerated process of articular cartilage degeneration. However, the exact mechanism of its molecular responses remains unknown [42].

RUNX2 is a critical transcription factors that regulating chondrocytes and osteoblasts differentiation [43]. Multiple studies suggested that FGFR1 signalling regulates the RUNX2 expression, both *in vivo* and in vitro. Altered articular chondrocytes mainly initiate the progression of OA due to the damaged chondrocytes towards cartilage-degrading enzymes and inflammatory cytokines. The combination of these cytokines conceives the infiltration of phagocytic cells within the joints [44,45]. The upregulation of RUNX2 expression is highly associated with chondrocytes hypertrophy which is strongly correlated with the pathogenesis of OA [43,46].

Another pathway is P38 MAPK signalling pathway holds a significant role throughout several diseases, particularly for the initiation and progression of OA [47–49]. The release of MMPs, chemokines, COX-2 enzymes in human articular cartilage, and proinflammatory cytokines might be triggered by the activation of p38 MAPK pathways signalling [49]. Many experiments had tried to suppress the activation of the p38 MAPK signalling pathway in order to study the potential downregulation of inflammatory cells recruitment. The stoppage of this pathway tends to diminish the production of proinflammatory cytokines and apoptosis of chondrocytes in articular cartilage [50]. TNFα and IL-1β are the proinflammatory cytokines that had shown to be induced by the activation of the P38 MAPK pathway [51].

These pathways push a progressive change towards the pathophysiological knee of OA condition as the involvement of FGFR1 expressions increased. FGFR1 was found to be striving the catabolic responses resulting in the increase of a disintegrin and metalloproteinase with a thrombospondin type 1 motifADAMTS5 gene and matrix metalloproteinases 13 (MMP-13) [42]. Up-regulation in such enzymes were missing in the inactivation of FGFR1 signalling experiments [42,52]. The rise of FGFR1 signalling in the knee joint’s articular cartilage can also be reflected as the FGFR3 expression is gradually declined in OA patients. This decreased FGFR3 expression happens due to the FGF-2/FGFR1 signalling, which is found to be significantly increased [53].

Another important aspect in maintaining and regulating the articular cartilage that was recently discovered is autophagy [54]. Excessive autophagic situation can, later on, develop into a worse progression of OA. The autophagy activity is inhibited by the down-regulation of FGFR1 expression, although the precise details for thismechanism remain unclear [42,54,55]. The autophagic activity had been studied in vitro *and in vivo*. The microtubule-associated protein 1 light chain 3α (LC3) which is a marker for autophagosome showed an increased along with more expression of FGFR1 signalling. Later on, LC3 will convert to LC3-II as the marker for autophagic activity. Examination of this marker with Western Blotting showed an increase of 2.5 times from the normal LC3-II level [23]. A study in mice models showed that the deletion of FGFR1 expression was proven to be having a better aggrecan compared with mice with high FGFR1 expression [56]. Another study also reported the diminished amount of FGFR1 could delay the progression of OA in temporomandibular joint model [42].

### 3.3. Extracellular vesicle in osteoarthritis

EV is composed of variative micro- and nano-sized particles produced by both healthy and altered cells. EV is collectively classified as microparticle/microvesicles, exosomes, and apoptotic bodies [57,58]. Many studies have been conducted on the involvement of extracellular vesicles (EV) in osteoarthritis. Recently, it was revealed that EVs can also be generated from MSCs, and that they may have a wide range of therapeutic applications in a variety of human illnesses [59]. A number of studies have found that utilizing MSC-derived EVs to enhance cartilage repair and protect against OA-induced cartilage degradation has shown beneficial results [59–61]. Moreover, exosome release is limited by FGFR inhibitors and affect the stromal cells’ capacity to defend in OA [62].

EVs have the potential to deliver effects to specific cell types through ligand-receptor interactions [63]. Research involving the use of synthetic receptor-bound vesicles that binds to natural ligand was conducted as proof-of-concept that this therapeutic approach successfully depletes the targeted ligand by promoting its endocytic uptake and lysosomal degradation [64–66]. EVs are generally simple to operate and have a wide range of surface engineering as well as encapsulation capabilities. Molecules linked to the EV surface have been demonstrated to confer targeting ability, boost expression levels, improve solubility, and activate antigen immunogenicity, and they are predicted to have therapeutic benefits against different degenerative illnesses [67]. By providing an alternative receptor to selectively bind with the endogenous FGF1, this might have comparable effects in preventing future OA degeneration.

The pathogenesis of OA is complicated, and the involvement of many distinctive cells are difficult to be studied, especially the role of EVs. However, some cell types within synovial fluid like bone, ligaments, tendon, fibroblast-like synoviocyte and chondrocytes are acknowledged to produce abundant EVs [68]. EVs have been known to maintain the communication between distinct cells lineage. EVs held a crucial role in maintaining joint homeostasis by regulating ECM production, inflammatory responses, and cell proliferation [69,70]. It is found that OA-like situations in the chondrocytes might drive EVs to become more harmful and aggravate the OA condition [71]. ECM has a low cell density so that it holds a crucial role in articular cartilage properties. To maintain healthy articular cartilage integrity, a composure between ECM breakdown and synthesis should be achieved. As in OA condition, there is an alteration in maintaining the harmony of ECM synthesis and breakdown [72,73]. Hence, the synthesis of ECM could no longer keep up with the breakdown that further will appear as the clinical symptoms, including osteophyte formation, pain, and joint space narrowing. The condition might be proven by isolating the EVs from synovial fibroblast under the IL-1β condition, which shows an excess of degradable mediators. EVs product from the synovial joint itself may play significant roles in tissue repair and the recruitment of Mesenchymal stem-cell (MSCs), which further will be discussed in the other chapter [74].

Despite the indistinct explanation of the detailed mechanism, the properties of MMPs are considered to lead the ECM breakdown process. Specifically, MMP-13 is believed as the mediator accountable for the significant breakdown of ECM. In addition, the activation and production of such proteolytic enzymes could further trigger the production of IL-1β and TNF-α. As the degradation of cartilage progress, further induction of these proinflammatory cytokines might be generated through the autocrine mechanism, followed by distinctive proinflammatory cytokines, including IL-8 and IL-6 [75].

The breakdown of ECM can be seen as the most symptoms in OA are composed of both fibroblast-like synoviocytes and chondrocytes [76]. EVs particles confined from the synovial fibroblasts along with the administrationof IL-1β to imitates the pathological OA environment were proven to promote aggrecan and MMP-13 expression within the chondrocytes, indicating the process of tissue breakdown and degeneration [77].

Another known component of EVs is miRNA. miRNA is the non-coding group of single-stranded RNA, which consisted of 19–24 nucleotides [78]. In OA cases, some miRNAs were noticed to be increased along with a notable decrease in several miRNAs. miR-504-3p, miR-210-5p, miR-155-3p, and miR-16-2-3p are the upregulated miRNAs in the synovial fluid of OA patients. On the other hand, miR-6878-3p, miR-146a-5p, and miR-26a-5p are the miRNAs that were found to be downregulated [79]. These downregulated particles are linked with the process of mucin-type O-glycan biosynthesis, glycan degradation, and cell adhesion molecules [78–80]. miRNAs that were upregulated were associated with the metabolism of biotin and synthesis of thyroid hormone. Chondrocytes proliferation and apoptosis, regulated by glycogen synthase kinase-3β, can be altered following the expression of miR-372-3p [80]. The imbalance level of miRNAs may further worsen the degeneration of the cartilage. However, targeting miRNAs associated with signalling cascade may counter the activation of several proinflammatory cytokines pathways and avoid the occurrence of the disease [71].

Targeting the role of EVs to counter such inflammatory environment in OA conditions should be explored further. However, there has not been any perfect procedure or method to prepare and select EVs that will be administered. To acquire the best result of EVs, purification using ultracentrifugation followed by density gradient flotation needs to be done. The content of the EVs can be varied based on the origin of the tissue cultured [81]. To separate the chondrocytes, osteoblast, and adipocytes completely from MSCs, the procedure needs a change in the microenvironment that can be done by bioengineering. Later on, the EVs derived from MSCs can be administered as an injection into the joint space [82].

### 3.4. Extracellular vesicle interaction with FGFR1

The optimal OA management should concentrate on the repair of tissue homeostasis due to the strong biological fingerprint, rather than symptomatic [83]. EVs and embedded molecules, such as proteins, lipids, or nucleic acids, have been postulated in recent years as possible contributors to chondrocytes pro-regenerative and immunosuppressive capabilities, alongside secreted factors [83–85]. In fact, MSC-EVs have protective benefits on both chondrocytes and synoviocytes in OA patients by stimulating chondrocyte proliferation and migration [86,87]. Potential embedded molecules such as FGFR1-bound EVs, acting as natural FGFR1 competitor for ligand, have potentials in preventing the mineralization tidemark from migrating to the nonmineralized articular cartilage by inhibiting cartilage degeneration [88]. Moreover, a recent *in vivo* research targeting the natural receptor by providing an alternative synthetic ligand showed significantly reduction loss of proteoglycan and articular cartilage degradation, as well as the production of ECM degrading enzymes and death in articular chondrocytes [41]. Conversely, providing an alternative receptor with higher affinity to selectively bind to the natural ligand could possibly exert similar effects in preventing further degeneration in OA.

EVs are made up of a complex mixture of lipids, surface and membrane molecules, and other components; some of these components help in tissue targeting, while others maintain minimum non-specific interactions [89]. It was shown that nanobodies may be attached to the surface of extracellular vesicles using phospholipids, altering their cell targeting behavior at least in vitro [90]. Other researchers have achieved comparable findings by using native EV membrane proteins (e.g., Lamp2b and platelet-derived growth factor) as fusion partners in targeting ligands [91,92]. An N-terminal myristoylation signal (MYR) anchors artificial mem-opto-FGFR1 to the plasma membrane, followed by the KD, CTD, and LOV domains. mV-mem-opto-FGFR1 is inserted into the plasma membrane by incorporation of the transmembrane domain of p75 [93]. Another option is to genetically engineer vesicle-forming cells to make a transmembrane receptor or protein before vesicle formation, which has been extensively researched [94]. Furthermore, FGFR1 gene is amplifiable and dual-color silver-enhanced in situ hybridization could be used for assessing the amplification [95,96].

### 3.5. Reliability of the treatment in OA

EVs are essential biological microparticles that can prevent OA in numerous ways, particularly in its interaction with FGFR1 in human articular cartilage. Furthermore, MSC-EVs may provide specific interaction of the targeted tissues [89]. In addition, the usage of EVs is proven to suppress the MMP-13 expression, which is strongly correlated with the lower production of proinflammatory cytokines [42]. By reducing the production of some ECM degrading enzymes and maintaining the number of proteoglycans, EVs may prevent the progression of OA [41]. The ability to diminish the FGFR1 signalling would also suppress the autophagic infiltration in the articular cartilage and prevent further progression of OA. However, several pitfalls may also follow this novel treatment. There is no standardized procedure or validated method to isolate the specific origin of EVs [71]. The cost of such a procedure should also be calculated carefully as the multiple isolation methods may increase the cost, time, and effort. The complexity in developing the perfect isolation techniques may also be a burden towards establishing this therapy [97].

## 4. Conclusion

The most frequent causes of OA are underlying bone disease and the gradual degradation of the cartilage lining of one or more freely moving joints. The amount of FGFR1 is raised in OA degenerative cartilage, suggesting that FGFR1 is the primary FGF pathway in cartilage degeneration. Moreover, employment of MSC-EVs to improve cartilage repair and protect against OA-induced cartilage degradation has proved to be helpful in a number of trials. It results to the point that the novel usage of FGFR1-bound EV-derived MSC could be beneficial in the treatment of osteoarthritis by preventing ligation of FGF1 to the natural FGFR1. However, more clinical trials are needed to elucidate the specific clinical consequences and to understand the mechanism of this modality.
